# Autonomic dysfunction in muscular dystrophy: a theoretical framework for muscle reflex involvement

**DOI:** 10.3389/fphys.2014.00047

**Published:** 2014-02-18

**Authors:** Scott A. Smith, Ryan M. Downey, Jon W. Williamson, Masaki Mizuno

**Affiliations:** ^1^Department of Health Care Sciences, University of Texas Southwestern Medical CenterDallas, TX, USA; ^2^Internal Medicine, University of Texas Southwestern Medical CenterDallas, TX, USA

**Keywords:** sympathetic nerve activity, parasympathetic nerve activity, cardiovascular disease, muscle afferents, muscular dystrophy, exercise

## Abstract

Muscular dystrophies are a heterogeneous group of genetically inherited disorders whose most prominent clinical feature is progressive degeneration of skeletal muscle. In several forms of the disease, the function of cardiac muscle is likewise affected. The primary defect in this group of diseases is caused by mutations in myocyte proteins important to cellular structure and/or performance. That being stated, a growing body of evidence suggests that the development of autonomic dysfunction may secondarily contribute to the generation of skeletal and cardio-myopathy in muscular dystrophy. Indeed, abnormalities in the regulation of both sympathetic and parasympathetic nerve activity have been reported in a number of muscular dystrophy variants. However, the mechanisms mediating this autonomic dysfunction remain relatively unknown. An autonomic reflex originating in skeletal muscle, the exercise pressor reflex, is known to contribute significantly to the control of sympathetic and parasympathetic activity when stimulated. Given the skeletal myopathy that develops with muscular dystrophy, it is logical to suggest that the function of this reflex might also be abnormal with the pathogenesis of disease. As such, it may contribute to or exacerbate the autonomic dysfunction that manifests. This possibility along with a basic description of exercise pressor reflex function in health and disease are reviewed. A better understanding of the mechanisms that possibly underlie autonomic dysfunction in muscular dystrophy may not only facilitate further research but could also lead to the identification of new therapeutic targets for the treatment of muscular dystrophy.

## Introduction

Muscular dystrophies (MD) are a group of debilitating, incurable, and often lethal disorders that present with many of the same pathological features (Emery, 2002). A shared characteristic is the development of progressive skeletal muscle degeneration and weakness whose distribution can often be used to distinguish between the many forms of the disease (Duchenne, Becker, limb girdle, facioscapulohumeral, Emery-Dreifuss, myotonic dystrophy, etc.) (Emery, 1998). Involvement of respiratory muscles and the development of reduced ventilatory function is likewise a common feature with the distribution, progression, and severity varying greatly among the different dystrophies (Mercuri and Muntoni, 2013). Cardiac and smooth muscle can also be affected with the former manifesting as dilated cardiomyopathies and/or conduction defects and the latter presenting as problems with gastric emptying and/or urinary retention, for example (Yilmaz and Sechtem, 2012; Mercuri and Muntoni, 2013). The primary defect in the various muscular dystrophies involves mutations of myocyte proteins important to cellular structure and/or function. These include, but are not limited to, extracellular matrix and external membrane proteins, proteins associated with the sarcolemma, sarcomeric proteins, nuclear membrane proteins and enzymatic proteins (Mercuri and Muntoni, 2013). Despite this knowledge, the various factors that mediate the development of end-organ damage in the muscular dystrophies is not fully understood and represents an area of continued intense investigation. In this regard, the development of autonomic dysfunction in MD has received considerable attention recently.

## Autonomic dysfunction in muscular dystrophy

In addition to the primary muscular defects, another possible contributor to the generation of pathology in muscular dystrophy is the genesis of autonomic dysfunction. Impairments of the autonomic nervous system have been described in patients with Duchenne and Becker MD (Politano et al., 2008). In both types of MD, an autonomic imbalance has been observed in which sympathetic activity is significantly increased coupled with diminished parasympathetic activity (Yotsukura et al., 1995; Lanza et al., 2001; Inoue et al., 2009). It has been speculated that such an imbalance may contribute to the development of dilated cardiomyopathy, ventricular arrhythmias, and sudden cardiac death in these patients (Ducceschi et al., 1997). In the *mdx* mouse model of MD, a model mimicking the abnormality found in patients with Duchenne MD (i.e., deficiency of the cytoskeletal protein dystrophin), pharmacological autonomic blockade and baroreflex sensitivity testing have likewise demonstrated an imbalance in the autonomic regulation of heart rate being characterized by enhancements and decrements in sympathetic and parasympathetic activity, respectively (Chu et al., 2002). Similar findings have been reported in patients with facioscapulohumeral MD (DellaMarca et al., 2010). Autonomic abnormalities of varying degrees have likewise been reported in myotonic dystrophy (Inoue et al., 1995; DiLeo et al., 2004) as well as Emery-Dreifuss (Fujita et al., 2005), Fukuyama Congenital (Itoh et al., 1996), and Miyoshi MDs (Tomoda et al., 1994).

To date, most studies have focused on the impact autonomic dysfunction has on cardiac performance and morphology in MD with little attention given to its possible role in the development of skeletal myopathy (the primary pathology). Indeed, the autonomic nervous system is intimately involved in the control of blood flow to skeletal muscle via its modulation of cardiac output, blood pressure, and vascular resistance (Mitchell et al., 1994). Alterations in the ability of the autonomic nervous system to adequately regulate these hemodynamic variables could significantly compromise blood flow to peripheral skeletal muscle initiating and/or exacerbating the primary degenerative processes characteristic of the disease. As an example, normally during exercise sympathetic activity is increased while parasympathetic activity is largely withdrawn. As a result, cardiac output is enhanced and the resistance to blood flow in the visceral organs and non-contracting skeletal muscle is elevated. In contrast, sympathetic vasoconstriction is opposed in active skeletal muscle by local metabolic vasodilation (i.e., functional sympatholysis) augmenting blood flow (Remensnyder et al., 1962). In this way, autonomic modulation of the cardiovascular system closely matches the delivery of blood to meet the metabolic demands of the working muscle. In an elegant series of studies in patients with dystrophinopathies (Duchenne and Becker MD) as well as mouse models of the disorder, it has been repeatedly demonstrated that the metabolic modulation of sympathetic vasoconstriction in working skeletal muscle is impaired producing ischemia during exercise (Thomas et al., 1998; Sander et al., 2000; Lai et al., 2009; Martin et al., 2012). The studies indicate that this impaired function is largely due to the loss of sarcolemmal neuronal nitric oxide synthase which is requisite to produce the nitric oxide necessary to mitigate α-adrenergic vasoconstriction (Thomas et al., 1998; Sander et al., 2000). It has been suggested that repeated bouts of ischemia in these patients, which could occur in the performance of routine daily activities, may initiate and/or accelerate fiber necrosis in the muscle (Sander et al., 2000; Ito et al., 2006). Potentially, this could facilitate the early onset of muscle fatigue, limit exercise tolerance, and at the same time promote muscle degeneration (Thomas et al., 1998). Although these studies do not directly infer autonomic dysfunction but rather the inability to oppose sympathetic activity, they do demonstrate the potential impact abnormal alterations in sympathetic and parasympathetic activity during exercise may have on the regulation of skeletal muscle blood flow and, potentially, the generation of skeletal myopathy in MD.

## Autonomic regulation during exercise

Although in certain forms of MD physical activity is limited from birth, decrements in the ability to exercise present later (childhood, adolescence, adulthood) in other forms of the disease (Mercuri and Muntoni, 2013). In patients that do maintain the ability to perform exercise, even when limited, the development of autonomic dysfunction during physical activity could have a considerable impact on the generation of both cardiac and skeletal muscle abnormalities. Therefore, it is important to understand the mechanisms underlying the autonomic response to exercise. To this end, autonomic function is regulated by input from three distinct neural mechanisms during exercise: central command, the baroreflex (arterial and cardiopulmonary) and the exercise pressor reflex. Central command has been described as a feed-forward mechanism from higher brain centers involving the parallel activation of motor neurons for volitional movement and autonomic circuits within the brainstem (Goodwin et al., 1972). The baroreflex, with receptors located in the carotid sinuses and aortic arch (arterial baroreflex) as well as the heart and lungs (cardiopulmonary baroreflex), is a negative feedback system that modulates moment-to-moment variations in blood pressure by continually adjusting autonomic activity (Mancia and Mark, 1983; Mark and Mancia, 1983). The exercise pressor reflex (EPR) is a peripheral feedback input emanating from skeletal muscle that likewise transmits signals to autonomic centers within the brainstem (McCloskey and Mitchell, 1972). Collectively, these inputs mediate precise adjustments in sympathetic and parasympathetic activity that ensure appropriate changes in blood flow are made to meet the metabolic demands of working muscle. Given that the EPR originates in skeletal muscle and is likely affected by the degenerative processes that occur with MD, it is logical to suggest that this reflex could contribute to or exacerbate autonomic dysfunction during exercise in this disease.

## The skeletal muscle exercise pressor reflex

The EPR consists of two relatively distinct components. The afferent fibers of the first, termed the muscle mechanoreflex, consist predominately of thinly-myelinated group III (Aδ) neurons that terminate within collagen tissue between skeletal fibrocytes (Andres et al., 1985; Kaufman and Forster, 1996). Receptors activating these afferents are largely stimulated by changes in intra-muscular pressure as well as the mechanical distortion of the muscle during contraction (Stebbins et al., 1988; Williamson et al., 1994). As such, the afferents respond immediately (within 2–5 s) at the onset of physical activity (Kaufman et al., 1983). The associated receptors are not well defined but may include mechanogated potassium channels, *L* and *T*-type calcium channels and/or mechanogated cation channels (Hamill and McBride, 1996). The muscle metaboreflex is the second functional component of the EPR. The afferent fibers of this reflex are primarily composed of unmyelinated Group IV (C) neurons that terminate in the walls of capillaries, venules, and lymphatic vessels within skeletal muscle (Andres et al., 1985; Kaufman and Forster, 1996). These receptors are chemically-sensitive and stimulated by the metabolites produced during muscle work (Kniffki et al., 1978). As a result, activation of afferent fibers associated with these receptors is somewhat delayed (5–20 s) requiring sufficient time for the production and accumulation of chemical by-products by skeletal myocytes (Kaufman et al., 1983; Mense and Stahnke, 1983). Several chemicals have been shown to activate the metaboreflex including, but not limited to, lactic acid, potassium, bradykinin, diprotonated phosphate, analogs of adenosine triphosphate (ATP) and by-products of arachidonic acid metabolism (Rybicki et al., 1984; Stebbins and Longhurst, 1985; Rotto et al., 1989, 1990; Sinoway et al., 1994; Li et al., 2008). In addition, several receptors have been implicated in mediating activation of associated afferent fibers such as the ATP-gated ion-channel receptor (P2X_3_), the acid-sensing ion channel receptor (ASIC), the bradykinin receptor (B2), the transient receptor potential vanilloid 1 receptor (TRPv1), and the cannabinoid receptor (CB1) to name a few (Pan et al., 1993; Hanna et al., 2002; Li et al., 2004a; Williams et al., 2008; Smith et al., 2010). It should be noted that both groups of afferent fibers display a degree of polymorphism with some Group III neurons responding to chemical stimuli and some Group IV neurons responding to mechanical stimuli (Kaufman and Forster, 1996). In addition, evidence suggests that mechanically-sensitive afferents can be sensitized by metabolites (especially in the presence of low perfusion) whereas the activity of chemically-sensitive neurons is augmented during periods of muscle ischemia (Kaufman et al., 1984b).

Most group III and IV neurons first synapse in the dorsal horn of the spinal cord in Rexed's laminae I, II, V, and X with additional evidence suggesting dense projections to lamina VI in the rostral portion of the spinal cord (Kalia et al., 1981; Panneton et al., 2005). Several neurotransmitters and peptides are involved in conducting and modulating EPR signals in the spinal cord including glutamate, aspartate, substance P, and nitric oxide for example (Wilson et al., 1993; Hand et al., 1996; Li and Mitchell, 2002). Likewise, a number of receptors within the spinal cord are involved in this process to variable degrees including, but not limited to, NMDA receptors, non-NMDA receptors, NK-1 receptors, and P2X receptors (Wilson, 2000). Although not completely defined, from the dorsal horn muscle afferents travel along the dorsolateral sulcus and ventral spinal cord projecting to the cuneate nucleus, the nucleus tractus solitarius (NTS), the lateral reticular nucleus, the caudal ventrolateral medulla (CVLM), and the rostral ventrolateral medulla (RVLM) in the brain stem (Iwamoto et al., 1984; Kozelka and Wurster, 1985; Dykes and Craig, 1998; Potts et al., 2002). Evidence suggests that the primary processing center for this afferent information is within the NTS although several other areas may also contribute including the CVLM, RVLM, the lateral tegmental field, the nucleus ambiguus and the rostral periaqueductal gray (Iwamoto et al., 1982; Iwamoto and Kaufman, 1987; Person, 1989; Li et al., 1997; Ishide et al., 2000; Li and Mitchell, 2000). From the brainstem, parasympathetic preganglionic neurons with origins in the nucleus ambiguus travel to postganglionic neurons within the walls of the heart (Mendelowitz, 1999). Stimulation of the EPR decreases the activity of vagal motor neurons which serves to increase heart rate. Sympathetic premotor neurons project from the brainstem to preganglionic sympathetic neurons in the intermediolateral cell columns of the spinal cord which synapse in the paraveterbral chain ganglia. Transmission of this sympathetic signal continues to postganglionic neurons that innervate the heart and vasculature (Dampney et al., 2003). When activated, the EPR increases sympathetic nerve activity to the heart increasing its rate and contractile ability resulting in augmentations in cardiac output. Likewise, sympathetic activity to the arterial and venous circulations is concomitantly elevated mediating vasoconstriction. In short, the EPR induces autonomic adjustments to exercise by increasing sympathetic activity and largely withdrawing parasympathetic activity.

Key factors influence the magnitude of the autonomic and cardiovascular adjustments mediated by the EPR. For example, the type of muscle contraction performed effects the reflex responses produced. In general, relative tetanic contraction of skeletal muscle (e.g., static exercise) evokes a much larger increase in sympathetic activity, blood pressure, and heart rate than rhythmic contraction (e.g., dynamic exercise) (Perez-Gonzalez, 1981; Kaufman et al., 1984a). In addition, the greater tension developed in the muscle, the greater the expression of the EPR (Iwamoto and Botterman, 1985). The amount of muscle mass engaged likewise impacts the size of the response. In animals and humans, it has been demonstrated on several occasions that the larger the muscle mass contracted, the larger the sympathetically-mediated cardiovascular response elicited (McCloskey and Steatfield, 1975; Iwamoto and Botterman, 1985; Iellamo et al., 1999). The fiber type of the skeletal muscle contracted also plays a role in determining the size of the evoked response. A seminal study in rabbits has demonstrated that the experimental conversion of the predominately glycolytic gastrocnemius muscle to a more oxidative fiber type significantly effects the expression of the EPR (Wilson et al., 1995). In the investigation, contraction of the converted gastrocnemius muscle did indeed elicit elevations in blood pressure. However, these elevations were smaller in magnitude than those evoked by contraction of the un-converted gastrocnemius muscle. Thus, although contraction of Type I slow-twitch oxidative fibers increases blood pressure it does so to a lesser extent than contraction of Type II fast-twitch glycolytic fibers.

## Exercise pressor reflex dysfunction in disease

There is precedent for development of EPR dysfunction with the pathogenesis of disease. For example, abnormalities in EPR-mediated autonomic control have been described in hypertension. Using a rat model of essential hypertension, it has been demonstrated on several occasions that preferential stimulation of the EPR elicits augmented elevations in heart rate and blood pressure resulting from exaggerated increases in sympathetic activity (Smith et al., 2006; Mizuno et al., 2011a,b). Further research has delineated that this autonomic sympathetic dysfunction is mediated by overactivity of both functional components of the EPR (i.e., the muscle mechanoreflex and metaboreflex) (Leal et al., 2008). The mechanisms underlying EPR overactivity in this model of hypertension remain relatively unclear although current evidence suggests that abnormalities in the expression and/or sensitivity of skeletal muscle mechanoreceptors and/or metaboreceptors may contribute significantly. Blockade of mechanoreceptors with the trivalent lanthanide gadolinium substantially mitigates the abnormally enhanced increases in sympathetic activity, blood pressure, and heart rate in response to EPR stimulation in hypertensive rats (Mizuno et al., 2011b). Likewise, antagonism of the TRPv1 receptor (associated with the muscle metaboreflex) with capsazepine has similar effects (Mizuno et al., 2011a). TRPv1 protein expression has also been reported to be upregulated in the dorsal root ganglia subserving the afferent fibers of the EPR (Mizuno et al., 2011a). Alterations in the central processing of EPR afferent information may also contribute. Recent studies have shown that blocking nitric oxide production in the NTS (nitric oxide normally serves to buffer EPR activity) of normotensive rats recapitulates the mechanoreflex overactivity manifest in hypertensive animals (Leal et al., 2012). In contrast, pharmacologically increasing nitric oxide production within the NTS of hypertensive rats abrogates mechanoreflex dysfunction (Leal et al., 2013). As a corollary finding, neuronal nitric oxide synthase expression (the enzyme responsible for producing nitric oxide) has been shown to be reduced in areas of the NTS excited by skeletal muscle reflex input in hypertensive animals (Murphy et al., 2013). Collectively, these studies suggest that the development of both central (brainstem) and peripheral (skeletal muscle) abnormalities contribute to EPR mediated autonomic dysfunction in essential hypertension. Interestingly, EPR dysfunction has been similarly described in other models of this disease such as prenatally programmed hypertension and angiotensin II induced hypertension (Koba et al., 2013; Mizuno et al., 2013). With regard to the latter, increases in oxidative stress within the skeletal muscle have been shown to evoke EPR overactivity suggesting yet another peripheral mechanism by which this reflex may become dysfunctional (Koba et al., 2013). Perhaps most importantly, exaggerations in EPR activity (specifically the metaboreflex) have recently been described in hypertensive patients verifying the pathogenesis of this disorder in humans (Sausen et al., 2009; Delaney et al., 2010). Reports demonstrating that hypertensive individuals have a larger percentage of Type II skeletal muscle fibers as compared to Type I likely contributes significantly to the development of EPR overactivity in these patients (Juhlin-Dannfelt et al., 1979).

It is well established that chronic hypertension can induce pathological hypertrophic cardiac remodeling leading to heart failure (Takimoto et al., 2005). Given this interrelationship, it is not surprising that autonomic regulation by the muscle reflex is likewise abnormal in this disease (Piepoli et al., 1999; Middlekauff et al., 2000; Negrao et al., 2001). For example, several studies have demonstrated that preferential stimulation of the EPR in rats with dilated cardiomyopathy evokes exaggerated increases in sympathetic activity resulting in abnormally large elevations in heart rate and blood pressure (Smith et al., 2003; Koba et al., 2008). What is surprising, however, is that the etiology of EPR overactivity in heart failure appears to be different than in hypertension. As described previously, in hypertension both the mechanically and chemically sensitive components of the EPR are inappropriately augmented. In heart failure, evidence in both animals and humans suggests that EPR overactivity is primarily driven by potentiation of the mechanoreflex whereas metaboreflex function may be blunted (Sterns et al., 1991; McClain et al., 1993; Middlekauff et al., 2001; Wang et al., 2010). With regard to the mechanoreflex, selective activation of muscle mechanoreceptors elicits enhanced increases in sympathetic activity (Li et al., 2004b; Smith et al., 2005a; Wang et al., 2010). Research suggests this may be due to a sensitization of the mechanoreceptors by the metabolites produced during muscle contraction (Middlekauff and Chiu, 2004; Gao et al., 2007; Koba et al., 2010; Wang et al., 2010). In support of this tenet, blockade of the B2 bradykinin receptor as well as the ATP P2X_3_ receptor attenuates the cardiovascular response to EPR and mechanoreflex activation to a greater extent in cardiomyopathic rats as compared to healthy controls (Koba et al., 2010; Wang et al., 2010). Further, P2X_3_ protein expression is enhanced in the dorsal root ganglion subserving mechano-sensitive afferents in heart failure animals (Gao et al., 2007; Wang et al., 2010). Similarly, antagonizing the enzyme cyclo-oxygenase 2 (COX-2), responsible for the production of prostaglandins from arachidonic acid, reduces the sympathetic response to stimulation of the mechanoreflex to a larger degree in heart failure rats and patients than in healthy controls (Middlekauff et al., 2008; Morales et al., 2012). COX-2 protein expression is likewise elevated in skeletal muscle of cardiomyopathic rats (Morales et al., 2012). In contrast, the cardiovascular response to administration of metaboreceptor agonists is attenuated in heart failure (Li et al., 2004b; Smith et al., 2005b). Interestingly, mRNA and protein expression for the TRPv1 receptor (a marker of chemically-sensitive group IV afferent fibers) is reduced in both the dorsal root ganglion and soleus muscle of heart failure rats (Smith et al., 2005b; Wang et al., 2010). Moreover, selective ablation of group IV afferent neurons in healthy rats has been shown to recapitulate the EPR overactivity that develops in heart failure (Smith et al., 2005b). These findings suggest that the withdrawal and/or de-sensitization of chemically-sensitive afferent fibers may contribute to the EPR dysfunction that manifests in heart failure but do not themselves drive the reflex's overactivity. These changes may occur as a result of the skeletal myopathy (conversion from Type I to Type II skeletal muscle fibers, skeletal muscle atrophy, compromised oxidative capacity) known to develop with the pathogenesis of heart failure (Lipkin et al., 1988). Increases in oxidative stress within skeletal muscle have likewise been implicated in the generation of EPR overactivity in this disease and may play an important role in the differential mechanoreflex and metaboreflex dysfunction that manifests (Koba et al., 2009).

## Exercise pressor reflex dysfunction in muscular dystrophy?

**Figure 1:**
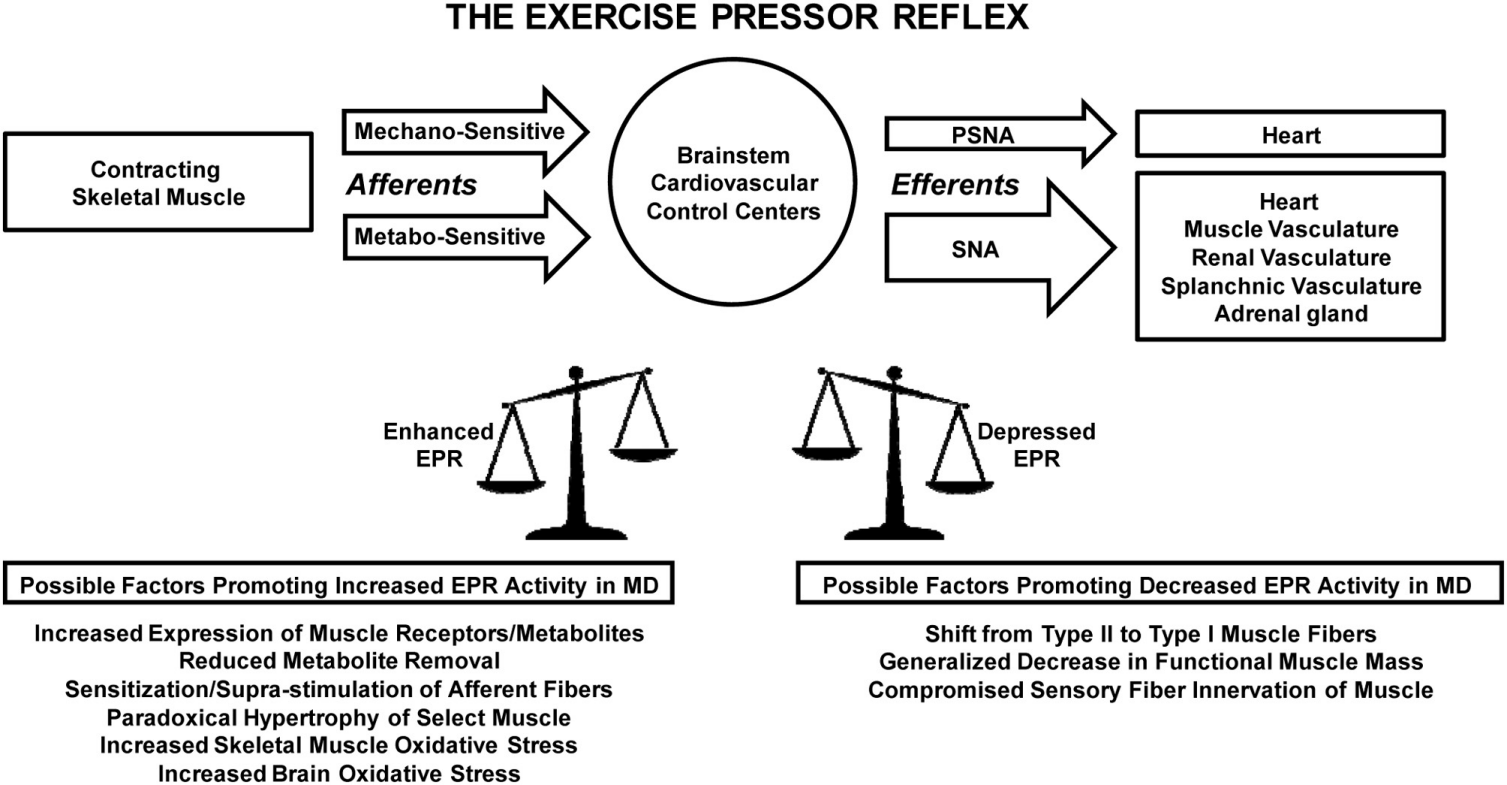
**Theoretical model of factors that could influence exercise pressor reflex function in muscular dystrophy**. During muscle contraction, the exercise pressor reflex is engaged upon stimulation of receptors which activate mechanically and metabolically sensitive afferent fibers innervating skeletal muscle. Sensory signals from these fibers are processed within autonomic control centers in the brainstem evoking decreases in parasympathetic nerve activity to the heart and increases in sympathetic nerve activity to the heart, adrenal gland, and vasculature of skeletal muscle, kidneys, and the splanchnic region. Numerous changes that occur with the pathogenesis of muscular dystrophy maintain the potential to both enhance and depress exercise pressor reflex activity. EPR, exercise pressor reflex; MD, muscular dystrophy; PSNA, parasympathetic nerve activity; SNA, sympathetic nerve activity.

Whether the EPR is abnormal in MD as it is in hypertension and heart failure remains to be determined. To our knowledge, there have been no studies conducted examining the function of this reflex in the muscular dystrophies. That being stated, clearly the functional anatomy of the EPR as well as the pathophysiology it displays in cardiovascular disease demonstrates its susceptibility to dysfunction with the advent of MD. In support of this concept, other causes of muscle degeneration such as disuse atrophy have been shown to alter muscle reflex function in both animals and humans (Kamiya et al., 2004; Hayashi et al., 2005). Further, a number of documented alterations that manifest with MD have the potential to directly influence EPR function. For example, similar to heart failure, increased expression of COX-2 and P2X protein has been demonstrated in skeletal muscle of *mdx* mice (Yeung et al., 2006; de Oliveira et al., 2013). As in both hypertension and heart failure, studies suggest that oxidative stress is likewise enhanced within skeletal muscle in MD and may also be increased in the brain; the latter of which could potentially affect central EPR processing (Kaczor et al., 2007; Sabharwal and Chapleau, 2014). As previously discussed, functional sympatholysis has been shown to be impaired in MD producing muscle ischemia during exercise (Thomas et al., 1998). As a result, removal of exercise-induced metabolites is likely compromised. This could potentially sensitize mechanically-sensitive afferent neurons (perhaps through P2X and COX-2 pathways as in heart failure) and/or augment the activity of metabolically-sensitive sensory fibers. Collectively, these changes would favor the generation of EPR overactivity. Such overactivity could be produced by the pathogenesis of EPR dysfunction or simply evoked by overstimulation of a normally operating reflex during ischemic exercise. Changes in muscle mass could also play a significant role. As previously stated, in general MD produces widespread and profound muscle atrophy (Kornegay et al., 2012). A loss of functional muscle mass would be expected to decrease expression of the EPR. That being stated, some muscle is spared in most forms of the disease and, in certain variations, a paradoxical hypertrophy often develops in select muscles (e.g., gastrocnemius in Duchenne and Becker MD) (Kornegay et al., 2012). As such, expression of the reflex would likely be dependent on the muscle being contracted and the extent to which it had been affected by the disease process. Unlike hypertension and heart failure, Type II fast-twitch fibers have been shown to be preferentially affected by the dystrophic process in certain forms of MD (e.g., Duchenne MD, facioscapulohumeral MD) displaying the earliest and most pronounced deterioration (Pedemonte et al., 1999; D'Antona et al., 2007). Type I slow-twitch fibers are affected secondarily and there is often a shift from fast-twitch to slow-twitch fibers (Webster et al., 1988; D'Antona et al., 2007). This too would be expected to depress EPR activity. Similarly, given the structural myocyte defects that develop with MD, it is possible that skeletal muscle innervation by Group III and IV sensory neurons is compromised in the disease. To date, little is known with regard to this possibility although motor innervation of the muscle has been shown to display longitudinal displacement with some axons ending freely in connective tissue rather than muscle fibers (Coers and Telerman-Toppet, 1977). It should be noted that the function of Group I and II sensory afferents (e.g., muscle spindles) appears to be preserved in MD (Aimonetti et al., 2005). Whether the same is true of Group III and IV fibers remains to be determined. A theoretical depiction of the factors that could affect EPR activity in MD is presented in Figure 1.

Although largely speculative at this point, alterations in EPR-mediated autonomic function in MD could significantly impact the progression of the disease. Again, metabolic modulation of sympathetically-mediated vasoconstriction during exercise is significantly diminished in certain forms of MD (i.e., impaired functional sympatholysis). This inability to buffer α-adrenergic vasoconstriction within the skeletal muscle would only be exacerbated by EPR-mediated exaggerations in sympathetic activity. This may contribute to the muscle ischemia that develops during exercise in these patients promoting tissue degeneration. Likewise, enhanced sympathetic activity to the heart evoked by the EPR could increase the risk for arrhythmogenesis, ventricular arrhythmia, and sudden death. As evidence, it has been demonstrated in rats that experimentally increasing sympathetic input to the heart can produce lethal cardiac arrhythmias (Oppenheimer et al., 1991). Prolonged EPR dysfunction could also play a significant role in the development of the cardiomyopathy that is a common clinical feature in many muscular dystrophies. Conversely, if EPR function is diminished in MD, as is its metabolic component in heart failure, the prognosis for EPR-induced pathology may be similar although the mechanisms slightly different. Decreases in EPR function could prevent the normal vasoconstrictor responses that occur in vascular beds not involved with exercise (e.g., visceral organs, non-exercising skeletal muscle). Vasoconstriction in these beds is important for re-directing blood flow to active skeletal muscle. The net effect of such an occurrence would be much the same as when the EPR is overactive; the development of ischemia in exercising muscle. This inability to increase sympathetic activity to the heart could likewise prevent augmentations in cardiac output needed to support the adequate delivery of blood to the working muscle. It might also increase the risk for the development of bradyarrythmias which have been described in some forms of MD (e.g., myotonic dystrophy type 1) (Aminoff et al., 1985). Undoubtedly, there is a significant need for research designed to address these possibilities.

## Exercise training in muscular dystrophy

The benefits of exercise training are numerous and include strengthening of skeletal muscle. In addition, training has been shown to improve autonomic function in disease states in which skeletal myopathy develops. For example, aerobic exercise training has been shown to markedly improve autonomic regulation by the EPR in heart failure (Wang et al., 2012a,b). Clearly, MD patients would benefit from improvements in both muscle strength and autonomic function. However, due to the progressive degenerative nature of the skeletal myopathy that is the hallmark feature of muscular dystrophy, patients often remain relatively sedentary and are, on many occasions, counseled to avoid excess physical activity (Sveen et al., 2008). The rationale underlying this prescription of non-exercise is the increased probability of exercise-induced muscle damage which may accelerate the progression of the disease (Petrof, 1998). That being stated, several reports suggest that, under the right conditions, exercise can evoke beneficial effects in certain forms of MD. For example, low intensity exercise training in *mdx* mice (treadmill running at 9 m/min) has been shown to reduce muscle damage caused by lipid and protein oxidation (Kaczor et al., 2007). In patients with Becker MD, 12 weeks of cycling exercise at 65% maximal oxygen uptake (VO_2max_) has been shown to improve strength in cycling muscles as well as VO_2max_ without increasing markers of skeletal muscle damage (Sveen et al., 2008). Similar beneficial effects of cycling exercise without concomitant muscle injury have been reported in patients with facioscapulohumeral MD (Olsen et al., 2005), myotonic dystrophy (Orngreen et al., 2005), and limb-girdle type 2I MD (Sveen et al., 2007). Likewise both low and high intensity resistance training (knee extension, elbow flexion) have been shown to increase muscle strength and endurance while being largely well tolerated in both Becker and limb-girdle type 2 MD patients (Sveen et al., 2013). Collectively, these findings suggest that regular, supervised exercise training may be a safe beneficial therapeutic modality to use in the management of MD patients that maintains the potential to increase daily function. However, the prescription of exercise in MD remains controversial and warrants additional investigation. Perhaps, as has been hypothesized, there may be a threshold of exercise intensity that demarks a positive therapeutic intervention from a deleterious activity that hastens pathology (Kaczor et al., 2007). If further research determines that changes in EPR activity contributes to and/or exacerbates autonomic dysfunction in MD, this factor must be taken into account as well when prescribing exercise.

## Summary

The muscular dystrophies are a group of muscle-wasting disorders that reduce quality of life and often lead to premature death. In addition to skeletal muscle, MD is known to deleteriously alter cardiac function in many forms of the disease. Recent evidence suggests that, although not the primary cause, abnormal regulation of the autonomic nervous system may contribute to the development of cardiac conduction anomalies as well as cardiomyopathy in MD. It has been postulated that such autonomic dysfunction may likewise be a causative factor in the progression of skeletal myopathy in this disease. The EPR is intimately involved in mediating the autonomic adjustments necessary to ensure the proper delivery of blood to working skeletal muscle. Given that this reflex originates in skeletal muscle, it may be vulnerable to the development of dysfunction in MD. MD-induced alterations in EPR function could compromise the reflex's ability to adequately increase blood flow to skeletal muscle during physical activity producing ischemia. Repeated bouts of ischemia during exercise could initiate and/or accelerate muscle degeneration in MD patients. Precedence for the pathogenesis of EPR dysfunction has been established in both hypertension and heart failure (a disease state often accompanied by significant skeletal myopathy) lending support to the tenet that EPR dysfunction may likewise develop in MD.

### Conflict of interest statement

The authors declare that the research was conducted in the absence of any commercial or financial relationships that could be construed as a potential conflict of interest.

## References

[B1] Aimonetti J. Ribot-Ciscar E. Rossi-Durand C. Attarian S. Pouget J. Roll J. (2005). Functional sparing of intrafusal muscle fibers in muscular dystrophies. Muscle Nerve 32, 88–94 10.1002/mus.20335 15806551

[B2] Aminoff M. Beckley D. McIlroy M. (1985). Autonomic function in myotonic dystrophy. Arch. Neurol. 42, 16 10.1001/archneur.1985.04060010018007 3966879

[B3] Andres K. H. During M. V. Schmidt R. F. (1985). Sensory innervation of the Achilles tendon by group III and IV afferent fibers. Anat. Embryol. 172, 145–156 10.1007/BF00319597 4051191

[B4] Chu V. Otero J. Lopez O. Sullivan M. Morgan J. Amende I. (2002). Electrocardiographic findings in mdx mice: a cardiac phenotype of Duchenne muscular dystrophy. Muscle Nerve 26, 513–519 10.1002/mus.10223 12362417

[B5] Coers C. Telerman-Toppet N. (1977). Morphological changes of motor units in Duchenne's muscular dystrophy. Arch. Neurol. 34, 396–402 10.1001/archneur.1977.00500190030004 880064

[B6] Dampney R. A. Horiuchi J. Tagawa T. Fontes M. A. P. Potts P. D. Polson J. W. (2003). Medullay and supramedullary mechanisms regulating sympathetic vasomotor tone. Acta Physiol. Scand. 177, 209–218 10.1046/j.1365-201X.2003.01070.x 12608991

[B7] D'Antona G. Brocca L. Pansarasa O. Rinaldi C. Tupler R. Bottinelli R. (2007). Structural and functional alterations of muscle fibers in the novel mouse model of facioscapulohumeral muscular dystrophy. J. Physiol. 584, 997–1009 10.1113/jphysiol.2007.141481 17855756 PMC2277004

[B8] Delaney E. Greaney J. Edwards D. Rose W. Fadel P. Farquhar W. (2010). Exaggerated sympathetic and pressor responses to handgrip exercise in older hypertensive humans: role of the muscle metaboreflex. Am. J. Physiol. 299, H1318–H1327 10.1152/ajpheart.00556.2010 20802135 PMC2993192

[B9] DellaMarca G. Frusciante R. Scatena M. Dittoni S. Testani E. Vollono C. (2010). Heart rate variability in facioscapulohumeral muscular dystrophy. Funct. Neurol. 25, 211–216 21388582

[B10] de Oliveira F. Quintana H. Bortolin J. Gomes O. Liberti E. Ribeiro D. (2013). Cyclooxygenase-2 expression in skeletal muscle of knockout mice suffering Duchenne muscular dystrophy. Histochem. Cell Biol. 139, 685–689 10.1007/s00418-012-1056-7 23188550

[B11] DiLeo R. Rodolico C. De Gregorio C. Recupero A. Coglitore S. Annesi G. (2004). Cardiovascular autonomic control in myotonic dystrophy type 1: a correlative study with clinical and genetic data. Neuromuscul. Disord. 14, 136–141 10.1016/j.nmd.2003.11.002 14733960

[B12] Ducceschi V. Nigro G. Sarubbi B. Comi L. Politano L. Petretta V. (1997). Autonomic nervous system imbalance and left ventricular systolic dysfunction as potential candidates for arrhythmogenesis in Becker muscular dystrophy. Int. J. Cardiol. 59, 275–279 10.1016/S0167-5273(97)02933-1 9183043

[B13] Dykes R. Craig A. (1998). Control of size and excitability of mechanosensory receptive fields in dorsal column nuclei by homolateral dorsal horn neurons. J. Neurophysiol. 80, 120–129 9658034 10.1152/jn.1998.80.1.120

[B14] Emery A. E. (1998). The muscular dystrophies. Br. Med. J. 317, 991–995 10.1136/bmj.317.7164.991 9765171 PMC1114045

[B15] Emery A. E. (2002). The muscular dystrophies. Lancet 359, 687–695 10.1016/S0140-6736(02)07815-7 11879882

[B16] Fujita T. Shimizu M. Kaku B. Kanaya H. Horita Y. Uno Y. (2005). Abnormal sympathetic innervation of the heart in a patient with Emery-Dreifuss muscular dystrophy. Ann. Nucl. Med. 19, 411–414 10.1007/BF03027407 16164199

[B17] Gao Z. Xing J. Sinoway L. Li J. (2007). P2X receptor-mediated muscle pressor reflex in myocardial infarction. Am. J. Physiol. 292, H939–H945 10.1152/ajpheart.00911.2006 17012345

[B18] Goodwin G. M. McCloskey D. I. Mitchell J. H. (1972). Cardiovascular and respiratory responses to changes in central command during isometric exercise at constant muscle tension. J. Physiol. 226, 173–190 4263680 10.1113/jphysiol.1972.sp009979 PMC1331159

[B19] Hamill O. McBride D. (1996). The pharmacology of mechanogated membrane ion channels. Pharmacol. Rev. 48, 231–252 8804105

[B20] Hand G. A. Kramer G. L. Petty F. Ordway G. A. Wilson L. B. (1996). Excitatory amino acid concentrations in the spinal dorsal horn of cats during muscle contraction. J. Appl. Physiol. 81, 368–373 8828687 10.1152/jappl.1996.81.1.368

[B21] Hanna R. L. Hayes S. G. Kaufman M. P. (2002). Alpha, beta Methylene ATP elicits a reflex pressor response arising from muscle in decerebrate cats. J. Appl. Physiol. 93, 834–841 10.1152/japplphysiol.00237.2002 12183475

[B22] Hayashi N. Koba S. Yoshida T. (2005). Disuse atrophy increases the muscle mechanoreflex in rats. J. Appl. Physiol. 99, 1442–1445 10.1152/japplphysiol.00180.2005 15976366

[B23] Iellamo F. Massaro M. Raimondi G. Peruzzi G. Legramante J. (1999). Role of muscular factors in cardiorespiratory responses to static exercise: contribution of reflex mechanisms. J. Appl. Physiol. 86, 174–180 9887128 10.1152/jappl.1999.86.1.174

[B24] Inoue K. Ogata H. Matsui M. Hayano J. Miyake S. Kumashiro M. (1995). Assessment of autonomic function in myotonic dystrophy by spectral analysis of heart-rate variability. J. Auton. Nerv. Syst. 55, 131–134 10.1016/0165-1838(95)00040-5 8690846

[B25] Inoue M. Mori K. Hayabuchi Y. Tatar K. Kagami S. (2009). Autonomic function in patients with Duchenne muscular dystrophy. Pediatr. Int. 51, 33–40 10.1111/j.1442-200X.2008.02656.x 19371275

[B26] Ishide T. Mancini M. Maher T. J. Chayaikul P. Ally A. (2000). Rostral ventrolateral medulla opioid receptor activation modulates glutamate release and attenuates the exercise pressor reflex. Brain Res. 865, 177–185 10.1016/S0006-8993(00)02192-2 10821919

[B27] Ito K. Kimura S. Ozasa S. Matsukura M. Ikezawa M. Yoshioka K. (2006). Smooth muscle-specific dystrophin expression improves aberrant vasoregulation in mdx mice. Hum. Mol. Genet. 15, 2266–2275 10.1093/hmg/ddl151 16777842

[B28] Itoh M. Houdou S. Kawahara H. Ohama E. (1996). Morphological study of the brainstem in Fukuyama type congenital muscular dystrophy. Pediatr. Neurol. 15, 327–331 10.1016/S0887-8994(96)00230-5 8972533

[B29] Iwamoto G. Botterman B. (1985). Peripheral factors influencing expression of pressor reflex evoked by muscular contraction. J. Appl. Physiol. 58, 1676–1682 3997730 10.1152/jappl.1985.58.5.1676

[B30] Iwamoto G. Botterman B. Waldrop T. (1984). The exercise pressor reflex: evidence for an afferent pressor pathway outside the dorsolateral sulcus region. Brain Res. 292, 160–164 10.1016/0006-8993(84)90901-6 6697204

[B31] Iwamoto G. A. Kaufman M. P. (1987). Caudal ventrolateral medullary cells responsive to static muscular contraction. J. Appl. Physiol. 62, 149–157 3558174 10.1152/jappl.1987.62.1.149

[B32] Iwamoto G. A. Kaufman M. P. Botterman B. R. Mitchell J. H. (1982). Effects of lateral reticular nucleus lesions on the exercise pressor reflex in cats. Circ. Res. 51, 400–403 10.1161/01.RES.51.3.400 7116584

[B33] Juhlin-Dannfelt A. Frisk-Holmberg M. Karlsson J. Tesch P. (1979). Central and peripheral circulation in relation to muscle-fibre composition in normo-and hyper-tensive man. Clin. Sci. 56, 335–340 477218 10.1042/cs0560335

[B34] Kaczor J. Hall J. Payne E. Tarnopolsky M. (2007). Low intensity training decreases markers of oxidative stress in skeletal muscle of mdx mice. Free Radic. Biol. Med. 43, 145–154 10.1016/j.freeradbiomed.2007.04.003 17561103

[B35] Kalia M. Mei S. S. Kao F. F. (1981). Central projections from ergoreceptors (c fibers) in muscle involved in cardiopulmonary responses to static exercise. Circ. Res. 48, I48–I62 7226464

[B36] Kamiya A. Michikami D. Shiozawa T. Iwase S. Hayano J. Kawada T. (2004). Bed rest attenuates sympathetic and pressor responses to exercise in antigravity leg muscles in humans. Am. J. Physiol. 286, R844–R850 10.1152/ajpregu.00497.2003 14701716

[B37] Kaufman M. Rybicki K. Waldrop T. Mitchell J. (1984a). Effect on arterial pressure of rhythmically contracting the hindlimb muscle of cats. J. Appl. Physiol. 56, 1265–1271 6327583 10.1152/jappl.1984.56.5.1265

[B38] Kaufman M. P. Rybicki K. J. Waldrop T. G. Ordway G. A. (1984b). Effect of ischemia on responses of group III and IV afferents to contraction. J. Appl. Physiol. 57, 644–650 6092310 10.1152/jappl.1984.57.3.644

[B39] Kaufman M. P. Forster H. V. (1996). Reflexes controlling circulatory, ventilatory and airway responses to exercise, in Section 12, Exercise: Regulation and Integration of Multiple Systems, eds Rowell L. B. Shepherd J. T. (Bethesda, MD: American Physiological Society), 381–447

[B40] Kaufman M. P. Longhurst J. C. Rybicki K. J. Wallach J. H. Mitchell J. H. (1983). Effects of static muscular contraction on impulse activity of groups III and IV afferents in cats. J. Appl. Physiol. 55, 105–112 6309712 10.1152/jappl.1983.55.1.105

[B41] Kniffki K. D. Mense S. Schmidt R. F. (1978). Responses of group IV afferent units from skeletal muscle to stretch, contraction, and chemical stimulation. Exp. Brain Res. 31, 511–522 10.1007/BF00239809 658178

[B42] Koba S. Gao Z. Sinoway L. (2009). Oxidative stress and the muscle reflex in heart failure. J. Physiol. 587, 5227–5237 10.1113/jphysiol.2009.177071 19723775 PMC2790260

[B43] Koba S. Watanabe R. Kano N. Watanabe T. (2013). Oxidative stress exaggerates skeletal muscle contraction-evoked reflex sympathoexcitation in rats with hypertension induced by angiotensin II. Am. J. Physiol. 304, H142–H153 10.1152/ajpheart.00423.2012 23086992

[B44] Koba S. Xing J. Sinoway L. Li J. (2008). Sympathetic nerve responses to muscle contraction and stretch in ischemic heart failure. Am. J. Physiol. 294, H311–H321 10.1152/ajpheart.00835.2007 17965282

[B45] Koba S. Xing J. Sinoway L. Li J. (2010). Bradykinin receptor blockade reduces sympathetic nerve response to muscle contraction in rats with ischemic heart failure. Am. J. Physiol. 298, H1438–H1444 10.1152/ajpheart.00558.2009 20207818 PMC2867438

[B46] Kornegay J. Childers M. Bogan D. Bogan J. Nghiem P. Wang J. (2012). The paradox of muscle hypertrophy in muscular dystrophy. Phys. Med. Rehabil. Clin. N. Am. 23, 149–172 10.1016/j.pmr.2011.11.014 22239881 PMC5951392

[B47] Kozelka J. Wurster R. (1985). Ascending spinal pathways for somatoautonomic reflexes in the anesthetized dog. J. Appl. Physiol. 58, 1832–1839 4008403 10.1152/jappl.1985.58.6.1832

[B48] Lai Y. Thomas G. Yue Y. Yang H. Li D. Long C. (2009). Dystrophins carrying spectrin-like repeats 16 and 17 anchor nNOS to the sarcolemma and enhance exercise performance in a mouse model of muscular dystrophy. J. Clin. Invest. 119, 624–635 10.1172/JCI36612 19229108 PMC2648692

[B49] Lanza G. A. DelloRusso A. Giglio V. (2001). Impairment of cardiac autonomic function in patients with Duchenne muscular dystrophy: relationship to myocardial and respiratory function. Am. Heart J. 141, 808–812 10.1067/mhj.2001.114804 11320370

[B50] Leal A. Mitchell J. Smith S. (2013). Treatment of muscle mechanoreflex dysfunction in hypertension: effects of L-arginine dialysis in the nucleus tractus solitarii. Exp. Physiol. 98, 1337–1348 10.1113/expphysiol.2012.071563 23771911 PMC3750060

[B51] Leal A. Murphy M. Iwamoto G. Mitchell J. Smith S. (2012). A role for nitric oxide within the nucleus tractus solitarii in the development of muscle mechanoreflex dysfunction in hypertension. Exp. Physiol. 97, 1292–1304 10.1113/expphysiol.2012.065433 22581746 PMC3480555

[B52] Leal A. K. Williams M. A. Garry M. G. Mitchell J. H. Smith S. A. (2008). Evidence for functional alterations in the skeletal muscle mechanoreflex and metaboreflex in hypertensive rats. Am. J. Physiol. 295, H1429–H1438 10.1152/ajpheart.01365.2007 18641268 PMC2593527

[B53] Li J. Gao Z. Kehoe V. Xing J. King N. Sinoway L. (2008). Interstitial adenosine triphosphate modulates muscle afferent nerve-mediated pressor reflex. Muscle Nerve 38, 972–977 10.1002/mus.21014 18570238 PMC3600608

[B54] Li J. Hand G. A. Potts J. T. Wilson L. B. Mitchell J. H. (1997). c-Fos expression in the medulla induced by static muscle contraction in cats. Am. J. Physiol. 272, H48–H56 9038921 10.1152/ajpheart.1997.272.1.H48

[B55] Li J. Maile M. D. Sinoway A. N. Sinoway L. I. (2004a). Muscle pressor reflex: potential role of vanilloid type 1 receptor and acid-sensing ion channel. J. Appl. Physiol. 97, 1709–1714 10.1152/japplphysiol.00389.2004 15220301

[B57] Li J. Mitchell J. (2002). Role of NO in modulating neuronal activity in superficial dorsal horn of spinal cord during exercise pressor reflex. Am. J. Physiol. 283, H1012–H1018 10.1152/ajpheart.00174.2002 12181131

[B58] Li J. Mitchell J. H. (2000). c-Fos expression in the midbrain periaqueductal gray during static muscle contraction. Am. J. Physiol. 279, H2986–H2993 11087256 10.1152/ajpheart.2000.279.6.H2986

[B56] Li J. Sinoway A. N. Gao Z. Maile M. D. Pu M. Sinoway L. I. (2004b). Muscle mechanoreflex and metaboreflex responses after myocardial infarction in rats. Circulation 110, 3049–3054 10.1161/01.CIR.0000147188.46287.1B 15520319

[B59] Lipkin D. P. Jones D. A. Round J. M. Poole-Wilson P. A. (1988). Abnormalities of skeletal muscle in patients with chronic heart failure. Int. J. Cardiol. 18, 187–195 10.1016/0167-5273(88)90164-7 2830194

[B60] Mancia G. Mark A. L. (1983). Arterial baroreflexes in humans, in Handbook of Physiology The Cardiovascular System Peripheral Circulation and Organ Blood Flow, eds Shepherd J. T. Abboud F. M. (Bethesda, MD: American Physiological Society), 755–793

[B61] Mark A. L. Mancia G. (1983). Cardiopulmonary baroreflexes in humans, in Handbook of Physiology The Cardiovascular System Peripheral Circulation and Organ Blood Flow, eds Shepherd J. T. Abboud F. M. (Bethesda, MD: American Physiological Society), 795–813

[B62] Martin E. Barresi R. Byrne B. Tsimerinov E. Scott B. Walker A. (2012). Tadalafil alleviates muscle ischemia in patients with Becker muscular dystrophy. Sci. Transl. Med. 4, 162ra155 10.1126/scitranslmed.3004327 23197572 PMC3935430

[B63] McClain J. Hardy C. Enders B. Smith M. Sinoway L. (1993). Limb congestion and sypathoexcitation during exercise: implications for congestive heart failure. J. Clin. Invest. 92, 2353–2359 10.1172/JCI116840 8227351 PMC288417

[B64] McCloskey D. Steatfield K. (1975). Muscular reflex stimuli to the cardiovascular system during isometric contractions of muscle groups of different mass. J. Physiol. 250, 431–441 1177147 10.1113/jphysiol.1975.sp011063 PMC1348370

[B65] McCloskey D. I. Mitchell J. H. (1972). Reflex cardiovascular and respiratory responses originating in exercising muscle. J. Physiol. 224, 173–186 5039977 10.1113/jphysiol.1972.sp009887 PMC1331532

[B66] Mendelowitz D. (1999). Advances in parasympathetic control of heart rate and cardiac function. News Physiol. Sci. 14, 155–161 11390842 10.1152/physiologyonline.1999.14.4.155

[B67] Mense S. Stahnke S. (1983). Responses in muscle afferent fibers of slow conduction velocity to contractions and ischaemia in the cat. J. Physiol. (Lond.) 342, 383–397 6631740 10.1113/jphysiol.1983.sp014857 PMC1193965

[B68] Mercuri E. Muntoni F. (2013). Muscular dystrophies. Lancet 381, 845–860 10.1016/S0140-6736(12)61897-2 23465426

[B69] Middlekauff H. Chiu J. (2004). Cyclooxygenase products sensitize muscle mechanoreceptors in healthy humans. Am. J. Physiol. 287, H1944–H1949 10.1152/ajpheart.00329.2004 15475528

[B70] Middlekauff H. Chiu J. Hamilton M. Fonarow G. MacLellan W. Hage A. (2008). Cyclooxygenase products sensitize muscle mechanoreceptors in humans with heart failure. Am. J. Physiol. 294, H1956–H1962 10.1152/ajpheart.01304.2007 18296564

[B71] Middlekauff H. R. Nitzsche E. U. Hoh C. K. Hamilton M. A. Fonarow G. C. Hage A. (2001). Exaggerated muscle mechanoreflex control of reflex renal vasoconstriction in heart failure. J. Appl. Physiol. 90, 1714–1719 11299260 10.1152/jappl.2001.90.5.1714

[B72] Middlekauff H. R. Nitzsche E. U. Hoh C. K. Hamilton M. A. Gonarow G. C. Hage A. (2000). Exaggerated renal vasoconstriction during exercise in heart failure patients. Circulation 101, 784–789 10.1161/01.CIR.101.7.784 10683353

[B73] Mitchell J. H. Haskell W. L. Raven P. B. (1994). Classification of Sports. J. Am. Coll. Cardiol. 24, 864–866 10.1016/0735-1097(94)90841-9 7930217

[B74] Mizuno M. Murphy M. Mitchell J. Smith S. (2011a). Antagonism of the TRPv1 receptor partially corrects muscle metaboreflex overactivity in spontaneously hypertensive rats. J. Physiol. 589, 6191–6204 10.1113/jphysiol.2011.214429 22025666 PMC3286695

[B75] Mizuno M. Murphy M. Mitchell J. Smith S. (2011b). Skeletal muscle reflex-mediated changes in sympathetic nerve activity are abnormal in spontaneously hypertensive rats. Am. J. Physiol. 300, H968–H977 10.1152/ajpheart.01145.2010 21217062 PMC3064311

[B76] Mizuno M. Siddique K. Baum M. Smith S. (2013). Prenatal programming of hypertension induces sympathetic overactivity in response to physical stress. Hypertension 61, 180–186 10.1161/HYPERTENSIONAHA.112.199356 23150514 PMC3525329

[B77] Morales A. Gao W. Lu J. Xing J. Li J. (2012). Muscle cyclo-oxygenase-2 pathway contributes to the exaggerated muscle mechanoreflex in rats with congestive heart failure. Exp. Physiol. 97, 943–954 10.1113/expphysiol.2012.065425 22523381 PMC3606264

[B78] Murphy M. Mizuno M. Downey R. Squiers J. Squiers K. Smith S. (2013). Neuronal nitric oxide synthase expression is lower in areas of the nucleus tractus solitarius excited by skeletal muscle reflexes in hypertensive rats. Am. J. Physiol. 304, H1547–H1557 10.1152/ajpheart.00235.2012 23564306 PMC3680727

[B79] Negrao C. E. Rondon M. U. Tinucci T. Alves M. J. Roveda F. Braga A. M. (2001). Abnormal neurovascular control during exercise is linked to heart failure severity. Am. J. Physiol. 280, H1286–H1292 11179075 10.1152/ajpheart.2001.280.3.H1286

[B80] Olsen D. Orngreen M. Vissing J. (2005). Aerobic training improves exercise performance in facioscapulohumeral muscular dystrophy. Neurology 64, 1064–1066 10.1212/01.WNL.0000150584.45055.27 15781829

[B81] Oppenheimer S. Wilson J. Guiraudon C. Cachetto D. (1991). Insular cortex stimulation produces lethal cardiac arrhythmias: a mechanism of sudden death? Brain Res. 550, 115–121 10.1016/0006-8993(91)90412-O 1888988

[B82] Orngreen M. Olsen D. Vissing J. (2005). Aerobic training in patients with myotonic dystrophy type 1. Ann. Neurol. 57, 754–757 10.1002/ana.20460 15852373

[B83] Pan H. L. Stebbins C. L. Longhurst J. C. (1993). Bradykinin contributes to the exercise pressor reflex: mechanism of action. J. Appl. Physiol. 75, 2061–2068 8307860 10.1152/jappl.1993.75.5.2061

[B84] Panneton W. M. Gan Q. Juric R. (2005). The central termination of sensory fibers from nerves to the gastrocnemius muscle of the rat. Neuroscience 134, 175–187 10.1016/j.neuroscience.2005.02.032 15953682

[B85] Pedemonte M. Sandri C. Schiaffino S. Minetti C. (1999). Early decrease of IIx myosin heavy chain transcripts in Duchenne muscular dystrophy. Biochem. Biophys. Res. Commun. 255, 466–469 10.1006/bbrc.1999.0213 10049732

[B86] Perez-Gonzalez J. (1981). Factors determining the blood pressure responses to isometric exercise. Circ. Res. 48, 176–186 7014025

[B87] Person R. J. (1989). Somatic and vagal afferent convergence on solitary tract neurons in cat: electrophysiological characteristics. Neuroscience 30, 283–295 10.1016/0306-4522(89)90254-6 2747918

[B88] Petrof B. (1998). The molecular basis of activity-induced muscle injury in Duchenne muscular dystrophy. Mol. Cell. Biochem. 179, 111–123 10.1023/A:1006812004945 9543354

[B89] Piepoli M. Ponikowski P. Clark A. L. Banasiak W. Capucci A. Coats A. J. S. (1999). A neural link to explain the “muscle hypothesis” of exercise intolerance in chronic heart failure. Am. Heart J. 137, 1050–1056 10.1016/S0002-8703(99)70361-3 10347330

[B90] Politano L. Palladino A. Nigro G. Scutifero M. Cozza V. (2008). Usefulness of heart rate variability as a predictor of sudden cardiac death in muscular dystrophies. Acta Myol. 27, 114–122 19472920 PMC2858940

[B91] Potts J. T. Lee S. M. Anguelov P. I. (2002). Tracing of projection neurons from the cervical dorsal horn to the medulla with the anterograde tracer biotinylated dextran amine. Auton. Neurosci. 98, 64–69 10.1016/S1566-0702(02)00034-6 12144043

[B92] Remensnyder J. P. Mitchell J. H. Sarnoff S. J. (1962). Functional sympatholysis during muscular activity. Circ. Res. 11, 370–380 10.1161/01.RES.11.3.370 13981593

[B93] Rotto D. Stebbins C. Kaufman M. (1989). Reflex cardiovascular and ventilatory responses to increasing H+ activity in cat hindlimb muscle. J. Appl. Physiol. 67, 256–263 2759951 10.1152/jappl.1989.67.1.256

[B94] Rotto D. M. Hill J. M. Schultz H. D. Kaufman M. P. (1990). Cyclooxygenase blockade attenuates the responses of group IV muscle afferents to static contraction. Am. J. Physiol. 259, H745–H750 2118727 10.1152/ajpheart.1990.259.3.H745

[B95] Rybicki K. J. Kaufman M. P. Kenyon J. L. Mitchell J. H. (1984). Arterial pressure responses to increasing interstitial potassium in hindlimb muscle of dogs. Am. J. Physiol. 247, R717–R721 6437247 10.1152/ajpregu.1984.247.4.R717

[B96] Sabharwal R. Chapleau M. (2014). Autonomic, locomotor and cardiac abnormalities in a mouse model of muscular dystrophy: targeting the renin angiotensin system. Exp. Physiol. [Epub ahead of print]. 10.1113/expphysiol.2013.074336 24334334 PMC4322680

[B97] Sander M. Chavoshan B. Harris S. Iannaccone S. Stull J. Thomas G. (2000). Functional muscle ischemia in neuronal nitric oxide synthase-decicient skeletal muscle of children with Duchenne muscular dystrophy. Proc. Natl. Acad. Sci. U.S.A. 97, 13818–13823 10.1073/pnas.250379497 11087833 PMC17659

[B98] Sausen M. T. Delaney E. P. Stillabower M. E. Farquhar W. B. (2009). Enhanced metaboreflex sensitivity in hypertensive humans. Eur. J. Appl. Physiol. 105, 351–356 10.1007/s00421-008-0910-8 18989694

[B99] Sinoway L. I. Smith M. B. Enders B. Leuenberger U. (1994). Role of diprotonated phosphate in evoking muscle reflex responses in cats and humans. Am. J. Physiol. 267, H770–H778 8067433 10.1152/ajpheart.1994.267.2.H770

[B100] Smith S. Leal A. Williams M. Murphy M. Mitchell J. Garry M. (2010). The TRPv1 receptor is a mediator of the exercise pressor reflex in rats. J. Physiol. 599, 1179–1189 10.1113/jphysiol.2009.184952 20142275 PMC2853004

[B101] Smith S. A. Mammen P. P. A. Mitchell J. H. Garry M. G. (2003). Role of the exercise pressor reflex in rats with dilated cardiomyopathy. Circulation 108, 1126–1132 10.1161/01.CIR.0000084538.40542.56 12925464

[B102] Smith S. A. Mitchell J. H. Naseem R. H. Garry M. G. (2005a). Mechanoreflex mediates the exaggerated exercise pressor reflex in heart failure. Circulation 112, 2293–2300 10.1161/CIRCULATIONAHA.105.566745 16216976

[B104] Smith S. A. Williams M. A. Leal A. K. Mitchell J. H. Garry M. G. (2006). Exercise pressor reflex function is altered in spontaneously hypertensive rats. J. Physiol. 577, 1009–1020 10.1113/jphysiol.2006.121558 17023501 PMC1890389

[B103] Smith S. A. Williams M. A. Mitchell J. H. Mammen P. P. A. Garry M. G. (2005b). The capsaicin-sensitive afferent neuron in skeletal muscle is abnormal in heart failure. Circulation 111, 2056–2065 10.1161/01.CIR.0000162473.10951.0A 15851614

[B105] Stebbins C. L. Brown B. Levin D. Longhurst J. C. (1988). Reflex effects of skeletal muscle mechanoreceptor stimulation on the cardiovascular system. J. Appl. Physiol. 65, 1539–1547 3182517 10.1152/jappl.1988.65.4.1539

[B106] Stebbins C. L. Longhurst J. C. (1985). Bradykinin-induced chemoreflexes from skeletal muscle: implications for the exercise reflex. J. Appl. Physiol. 59, 56–63 3861607 10.1152/jappl.1985.59.1.56

[B107] Sterns D. A. Ettinger S. M. Gray K. S. Whisler S. K. Mosher T. J. Smith M. B. (1991). Skeletal muscle metaboreceptor exercise responses are attenuated in heart failure. Circulation 84, 2034–2039 10.1161/01.CIR.84.5.2034 1934378

[B108] Sveen M. Andersen S. Ingelsrud L. Blichter S. Olsen N. Jonck S. (2013). Resistance training in patients with limb-girdle and Becker muscular dystrophies. Muscle Nerve 47, 163–169 10.1002/mus.23491 23169433

[B109] Sveen M. Jeppesen T. Hauerslev S. Kober L. Krag T. Vissing J. (2008). Endurance training improves fitness and strength in patients with Becker muscular dystrophy. Brain 131, 2824–2831 10.1093/brain/awn189 18776212

[B110] Sveen M. Jeppesen T. Hauerslev S. Krag T. Vissing J. (2007). An effective and safe treatment for patients with LGMD2I. Neurology 68, 59–61 10.1212/01.wnl.0000250358.32199.24 17200494

[B111] Takimoto E. Champion H. Li M. Belardi D. Ren S. Rodriguez E. (2005). Chronic inhibition of cyclic gmp phosphodiesterase 5a prevents and reverses cardiac hypertrophy. Nat. Med. 11, 214–222 10.1038/nm1175 15665834

[B112] Thomas G. Sander M. Lau K. Huang P. Stull J. Victor R. (1998). Impaired metabolic modulation of alph-adrenergic vasoconstriction in dystrophin-deficient skeletal muscle. Proc. Natl. Acad. Sci. U.S.A. 95, 15090–15095 10.1073/pnas.95.25.15090 9844020 PMC24580

[B113] Tomoda A. Zhao J. Ohtani Y. Miike T. Uchino M. Higuchi I. (1994). Two patients with distal muscular dystrophy and autonomic nerve dysfunction. Brain Dev. 16, 65–70 10.1016/0387-7604(94)90116-3 8059932

[B114] Wang H. Li Y. Gao L. Zucker I. Wang W. (2010). Alteration in skeletal muscle afferents in rats with chronic heart failure. J. Physiol. 588, 5033–5047 10.1113/jphysiol.2010.199562 21041525 PMC3036195

[B115] Wang H. Li Y. Zucker I. Wang W. (2012a). Exercise training prevents skeletal muscle afferent sensitization in rats with chronic heart failure. Am. J. Physiol. 302, R1260–R1270 10.1152/ajpregu.00054.2012 22496362 PMC3378347

[B116] Wang H. Zucker I. Wang W. (2012b). Muscle reflex in heart failure: the role of exercise training. Front. Physiol. 3, 1–16 10.3389/fphys.2012.00398 23060821 PMC3464681

[B117] Webster C. Silberstein L. Hays A. Blau H. (1988). Fast muscle fibers are preferentially affected in Duchenne muscular dystrophy. Cell 52, 503–513 10.1016/0092-8674(88)90463-1 3342447

[B118] Williams M. A. Smith S. A. O'Brien D. E. Mitchell J. H. Garry M. G. (2008). The group IV afferent neuron expresses multiple receptor alterations in cardiomyopathic rats: evidence at the cannabinoid CB_1_ receptor. J. Physiol. 586, 835–845 10.1113/jphysiol.2007.140392 18063665 PMC2375614

[B119] Williamson J. W. Mitchell J. H. Olesen H. L. Raven P. B. Secher N. H. (1994). Reflex increase in blood pressure induced by leg compression in man. J. Physiol. 475, 351–357 8021841 10.1113/jphysiol.1994.sp020076 PMC1160385

[B120] Wilson L. (2000). Spinal modulation of the muscle pressor reflex by nitric oxide and acetylcholine. Brain Res. Bull. 53, 51–58 10.1016/S0361-9230(00)00308-7 11033208

[B121] Wilson L. B. Dyke C. K. Parsons D. Wall P. T. Pawelczyk J. A. Williams R. S. (1995). Effect of skeletal muscle fiber type on the pressor response evoked by static contraction in rabbits. J. Appl. Physiol. 79, 1744–1752 8594037 10.1152/jappl.1995.79.5.1744

[B122] Wilson L. B. Fuchs I. E. Matsukawa K. Mitchell J. H. Wall P. T. (1993). Substance P release in the spinal cord during the exercise pressor reflex in anaesthetized cats. J. Physiol. 460, 79–90 7683719 10.1113/jphysiol.1993.sp019460 PMC1175202

[B123] Yeung D. Zablocki K. Lien C. Jiang T. Arkle S. Brutkowski W. (2006). Increased susceptibility to ATP via alteration of P2X receptor function in dystrophic mdx mouse muscle cells. FASEB J. 20, 610–620 10.1096/fj.05-4022com 16581969

[B124] Yilmaz A. Sechtem U. (2012). Cardiac involvement in muscular dystrophy: advances in diagnosis and therapy. Heart 98, 420–429 10.1136/heartjnl-2011-300254 22311853

[B125] Yotsukura M. Sasaki K. Kachi E. Sasaki A. Ishihara T. Ishikawa K. (1995). Circadian rhythm and variability of heart rate in Duchenne-type progressive muscular dystrophy. Am. J. Cardiol. 76, 947–951 10.1016/S0002-9149(99)80267-7 7484837

